# Genomic patterns of structural variation among diverse genotypes of *Sorghum bicolor* and a potential role for deletions in local adaptation

**DOI:** 10.1093/g3journal/jkab154

**Published:** 2021-05-05

**Authors:** Kittikun Songsomboon, Zachary Brenton, James Heuser, Stephen Kresovich, Nadia Shakoor, Todd Mockler, Elizabeth A Cooper

**Affiliations:** 1 Department of Bioinformatics and Genomics, University of North Carolina at Charlotte, Charlotte, NC 28223, USA; 2 North Carolina Research Campus, Kannapolis, NC 28081, USA; 3 Department of Plant and Environmental Sciences, Clemson University, Clemson, SC 29634, USA; 4 Donald Danforth Plant Science Center, St. Louis, MO 63132, USA

**Keywords:** structural variation, Sorghum, k-mean clustering, local adaptation

## Abstract

Genomic structural mutations, especially deletions, are an important source of variation in many species and can play key roles in phenotypic diversification and evolution. Previous work in many plant species has identified multiple instances of structural variations (SVs) occurring in or near genes related to stress response and disease resistance, suggesting a possible role for SVs in local adaptation. Sorghum [*Sorghum bicolor* (L.) Moench] is one of the most widely grown cereal crops in the world. It has been adapted to an array of different climates as well as bred for multiple purposes, resulting in a striking phenotypic diversity. In this study, we identified genome-wide SVs in the Biomass Association Panel, a collection of 347 diverse sorghum genotypes collected from multiple countries and continents. Using Illumina-based, short-read whole-genome resequencing data from every genotype, we found a total of 24,648 SVs, including 22,359 deletions. The global site frequency spectrum of deletions and other types of SVs fit a model of neutral evolution, suggesting that the majority of these mutations were not under any types of selection. Clustering results based on single nucleotide polymorphisms separated the genotypes into eight clusters which largely corresponded with geographic origins, with many of the large deletions we uncovered being unique to a single cluster. Even though most deletions appeared to be neutral, a handful of cluster-specific deletions were found in genes related to biotic and abiotic stress responses, supporting the possibility that at least some of these deletions contribute to local adaptation in sorghum.

## Introduction

Genomic structural mutations are an important source of variation in many species and can play key roles in phenotypic diversification and evolution. Structural variations (SVs) have been well-studied in human genomics for many years because of their roles in human disease (Feuk *et al.* 2006; MacDonald *et al.* 2014; Escaramís *et al.* 2015), but until more recently, much less was known about the extent or importance of SVs in plant species (Saxena *et al.* 2014). Categories of SVs include deletions, insertions, tandem duplications, inversions, relocations, and translocations (Feuk *et al.* 2006). These types of mutations can encompass entire gene sequences or even multiple gene sequences, giving them the potential to effect high-impact phenotypic changes in a single event. Plant evolution, in particular, has a rich history of large-scale structural mutations and dynamic genome rearrangements (Wendel *et al.* 2016) that have contributed to the current species diversity and adaptive potential, so identifying SVs in plant genomes is of great interest to plant breeders, evolutionary biologists, conservationists, and phylogeneticists (Swanson-Wagner *et al.* 2010; Saintenac *et al.* 2011; Cook *et al.* 2012; Díaz *et al.* 2012; Żmieńko *et al.* 2014).

Previous work has identified multiple instances of SVs occurring in or near genes related to both biotic and abiotic stress resistance in several plant species. For example, 10 tandem copies of the *rhg-b* gene in soybean were found to confer resistance to nematodes (Cook *et al.* 2012). In *Arabidopsis thaliana*, metabolite specialization conferred by copy number variants (CNVs) suggested that these mutations were involved in local adaptation (Shirai *et al.* 2017; Shirai and Hanada 2019). In rice, one of the largest studies of SVs in plants to date identified over 1.5 million SVs across 3000 genomes and confirmed earlier observations that many of these SVs occurred in or near genes related to disease resistance and stress response (Fuentes *et al.* 2019). Another study of 521 maize lines uncovered over 80,000 polymorphic SVs among accessions of the same species (Yang *et al.* 2019). Importantly, this study also found that many of these SVs were not in strong linkage disequilibrium (LD) with surrounding single nucleotide polymorphisms (SNPs), so they would be undetectable by conventional variant calling pipelines.

Sorghum [*Sorghum bicolor* (L.) Moench] is a versatile and highly adaptable C4 grass species that has been bred for diverse purposes. Following its initial domestication near present-day Sudan approximately 10,000 years ago, cultivated sorghum spread to regions of eastern, western, and southern Africa, as well as to the Middle East, Indian subcontinent, and parts of Asia (Kimber 2000). As the species reached new geographic locations and environments, it underwent strong local adaptation, ultimately resulting in five morphologically distinct races (bicolor, durra, guinea, caudatum, and kafir), but it is unknown what role, if any, SVs may have played in sorghum’s diffusion and diversification. Previous work examining SVs in sorghum using only a handful of lines showed that there were 33,598 SVs that were polymorphic between sweet and grain-type sorghums (Zheng *et al.* 2011; Zhang *et al.* 2014), suggesting that SVs could underlie key phenotypic differences in this species.

In this study, we aimed to characterize the broader population-level pattern of SVs in sorghum using whole-genome resequencing data from 347 diverse genotypes collected from multiple geographic locations. Using the SVs we identified in these data, we examined (1) whether or not SVs in sorghum are under positive or negative selection, (2) the extent to which particular SVs, and large deletions in particular, are associated with the geographic origins of each genotype, and (3) if any regionally-specific nonsynonymous deletions occurred in genes with functions potentially involved in local adaptation.

## Materials and methods

### Plant material and DNA extractions

The Sorghum Bioenergy Association Panel (BAP) is a diversity panel comprised of 390 sorghum genotypes that were originally developed to accelerate the breeding of sorghum as a potential bioenergy crop (Brenton *et al.* 2016). This study was based on 347 sorghum genotypes from the BAP that included representatives of each of the five major races (bicolor, durra, guinea, caudatum, and kafir) (Supplementary Table S1). The geographic origin of each genotype was retrieved from the U.S. Department of Agriculture’s Germplasm Repository Information Network (GRIN: https://www.ars-grin.gov/). Coordinates for each geographic location were approximated by locating the city or region listed in the GRIN database in Google Maps (Google, n.d.). If no city or regional information was available in the database, then we used the coordinates of the geometric center of the origin country.

The samples were shotgun sequenced (150-bp paired-end) on an Illumina X10 instrument at the HudsonAlpha Institute for Biotechnology as part of the TERRA-REF project (http://terraref.org/). Individual samples were multiplexed and run on a total of 123 lanes, resulting in an average of 30X coverage per sample. A handful (∼12) samples were sequenced twice; for these samples, we randomly selected just one run of sequencing so that all of the samples we compared would have the same average read depth. Raw sequencing reads are available through the TERRA-REF project page of the CyVerse repository (http://datacommons.cyverse.org/browse/iplant/home/shared/terraref).

### Structural variant calling and filtering

The pipeline for calling SVs in the BAP was adopted from the svtools pipeline (Larson *et al.* 2019). Briefly, de-multiplexed sequence reads in FASTQ format for each individual were aligned to version 3.0.1 of the BTx623 reference genome (as downloaded from Phytozome v12.1.6: https://phytozome.jgi.doe.gov/pz/portal.html) using the program speedseq (Chiang *et al.* 2015). SVs were identified in each individual aligned BAM file using LUMPY (Layer *et al.* 2014) with default parameters. The resulting 347 SV files were then sorted and merged with svtools (Larson *et al.* 2019). A full tutorial of this process has been delineated by the authors of svtools, and can be found at https://github.com/hall-lab/svtools/blob/master/Tutorial.md. The merged vcf was then used to calculate a genotype for each individual at the variant positions resulting in a fully genotyped vcf file of each individual. CNVnator (Abyzov *et al.* 2011) was run within svtools in order to annotate the called variants based on copy number. Subsequently, svtools merged the genotyped and CNV-annotated vcf files to remove any redundant variants that were called by both programs. The resulting set of SVs (available at https://datadryad.org/stash/share/X7mka20BtXuACd20QbtenGqS-yoGVfKZOZNQQzfe2B0) was further filtered in R with custom code (all R scripts are available at https://github.com/skittikun/SVs_in_sorghum_BAP). A minimum read depth of 10 was required to call an individual genotype as nonmissing. The SV calls were filtered to (1) require a paired-end (p.e.) matching value more than 3, (2) require a precise breakpoint (flag PRECISE), (3) remove any generic rearrangements of unknown architecture (break-end: BND), and (4) remove variants smaller than 50 bp or larger than 100,000 bp. All sites with more than 20% missing data were filtered out prior to any further analysis. For all analyses except for the genetic diversity and site frequency spectrum calculations, sites with a minor allele frequency (MAF) less than 0.05 were also removed. For all of the deletions that passed our filters (7766), we used snpEff (Cingolani *et al.* 2012) to annotate the potential functional impact of each variant.

In order to validate the SVs we called in this study, we searched our final vcf file for positions that overlapped with known deletions that had been previously identified and characterized in sorghum. These included mutations in *dw3* (Sobic.007G163800) (Multani *et al.* 2003), *Dry* (Sobic.006G147400) (Zhou *et al.* 2018), *Tan2* (Sobic.002G076600) (Wu *et al.* 2019), and several flowering genes (Sobic.006G004400, Sobic.006G057866, and Sobic.009G257300) (Li *et al.* 2018) (Supplementary Table S2).

### Genetic diversity

To compare the genomewide pattern of genetic diversity with the distribution of SVs, we called SNPs from the same sequencing data set. The raw sequencing reads were first trimmed with Trimmomatic (Bolger *et al.* 2014) and then aligned to the BTx623 reference genome with Bowtie2 (Langmead and Salzberg 2012). Aligned reads were sorted, indexed, and de-duplicated with SAMtools (Li *et al.* 2009), and then SNPs were called via the variant calling protocol implemented by the Genome Analysis Toolkit (GATK) version 3.8 (McKenna *et al.* 2010). The SNP calls then were filtered to remove sites with more than 20% missing data via vcftools (Danecek *et al.* 2011).

Nucleotide diversity (π) was estimated in 500 kb sliding windows using the formula described by Begun *et al.* (2007). The 500 kb-window π was estimated separately for SNPs and SVs, and the correlation between the two estimates was calculated in each window using custom R scripts.

### Global site frequency spectrum

To calculate the global site frequency spectrum, for each SV we first determined the number of genotypes having the minor allele at that site, where the possible number ranged from 1 up to 173 (this being the maximum number of times an SV could occur and still be considered the minor allele). For each bin size from 1 to 173, we calculated the total number of deletions in that bin.

To calculate the expected site frequency spectrum, we first calculated Watterson’s estimator of θ ($\theta_{w}$) (Watterson 1975). Given a sample of *n* individuals (or 2*n* in a diploid) and *S_n_* segregating sites, $\theta_{w}$ is defined as:

$$\theta_{w}=\frac{S_{n}}{\sum_{j=1}^{n-1}\frac{1}{j}}$$

Based on this equation, we calculated the expected number of deletions in each bin as $\theta_{w}*\frac{1}{i}$, where *i* was the size of the bin. The calculation and plots were performed in R using custom code available at https://github.com/skittikun/SVs_in_sorghum_BAP. Both the expected and observed site frequency spectra were calculated for (1) all SV types deletions only, (2) duplications only (Figure 2), (3) duplications only, and (4) inversions only (Supplementary Figure S2).

### Linkage disequilibrium and population structure

Because the majority of the SVs that we were able to confidently identify in our data were deletions (22,359 out of 24,648), we chose to focus exclusively on this type of SV for the rest of our analyses. Pairwise LD between deletions was estimated as the correlation coefficient (*r*^2^), which was calculated using the “-r2” option in PLINK version 1.9 (Purcell *et al.* 2007). In order to convert the filtered deletion calls (7766 SVs) into a format that was readable by PLINK, the binary values of 0 (absent), 1 (present), and missing NA were transformed to AA, TT, and 00, respectively with custom R code. In order to compare the extent of LD surrounding SVs, the distance at which LD had decayed to half of its maximum value (LD_1/2_) was determined (Otyama *et al.* 2019). The pattern of LD decay was calculated separately for the complete set of filtered deletions, deletions within gene regions, and deletions only within coding sequences (CDS). The positions of gene regions and coding sequences were determined based on version 3.1.1 of the Sorghum BTx623 reference genome annotation, which was downloaded from Phytozome.

Population structure based on SNPs was estimated using k-means clustering as implemented in the R package “cluster” (Maechler *et al.* 2021). The optimal number of groups was determined via an average silhouette method (Kaufman and Rousseeuw 2009). This method determines the silhouette width of a given point from its cluster apart from the other clusters. By varying the number of clusters (k), the average silhouette width can be computed for each k. The highest width indicates the optimal value of k. A principal component analysis (PCA) was implemented via the R package “FactoMineR” (Lê *et al.* 2008). After determining the best *k* value from k-means clustering, the program ADMIXTURE was run with default parameters (Alexander *et al.* 2015) and a *k* equal to our optimal value in order to generate ADMIXTURE plots. The patterns observed in each method were then compared with known racial and geographic origins data for each genotype.

### Identifying cluster-specific deletions affecting genes

To find genomic regions with a significantly different distribution of deletions across the eight clusters identified by k-means clustering, we calculated the mean weighted abundance percentages of deletions among the eight clusters in 500 kb sliding windows across the genome. Chi-square tests were used to calculate the significance of each window. Differences in abundance were considered statistically significant if they had a Bonferroni corrected *P*-value < 0.05.

To identify the unique deletions (or nearly unique) to a particular cluster or population of sorghums for each of the 7766 deletions, we first calculated the percent of alternate alleles that were found in each of the eight clusters identified by k-means clustering. Next, we tested whether or not the observed percentages of each deletion were significantly different from what would be expected by chance using a Chi-square goodness-of-fit test as implemented in R. For sites with a statistically significant difference in deletion abundance among the clusters, we further restricted them to include only sites where a single cluster contained 70% or more of the alternate alleles. Finally, for these sites, we also conducted a permutation test to ensure that differences in cluster size were not sufficient to explain the differences in relative abundance. More specifically, for each permutation test, we randomly permuted the alleles 100 times, and counted how many permutations also exhibited a single cluster with 70% or more of the alternate alleles. If fewer than 5% of the permutations showed this pattern, then we considered the deletion to be cluster-specific.

As an example, one of the sites that we classified as cluster-specific was Chr03_53588484_53588905_DEL. The alternate allele at this site occurred in 1, 2, 1, 0, 2, 19, 0, and 1 genotypes in k-means groups 1, 2, 3, 4, 5, 6, 7, and 8, respectively. At this same position, there were a total of 32, 31, 31, 30, 35, 53, 44, and 30 nonmissing genotypes in each of the five clusters, so the expected percentages were calculated as 11.50, 11.25, 11.25, 10, 12, 19, 15, and 10%. The Chi-square goodness of fit test resulted in a *P*-value of 4.314 × 10^−9^, indicating a significant difference between the observed and expected percentages. In other words, the distributions of the deletion alleles among these eight clusters were significantly different. The percentage of deletion alleles found within each cluster were 4, 8, 4, 0, 8, 73, 0, and 4%, respectively, suggesting that this site was potentially specific to cluster 6. In 100 random permutations, there were zero instances where an abundance of 70% or more was observed in cluster 6 by chance, so we considered this site to be cluster-specific (Supplementary Figure S1).

To determine if the cluster-specific deletions affected gene functions, we considered only the deletions causing nonsynonymous mutations with high or moderate impacts from snpEff result. In this study, we used the “rice-defline” descriptions from the “Sbicolor_454_v3.1.1.annotation_info.txt” file downloaded from Phytozome to define the gene functions. We additionally filtered our list of genes affected by cluster-specific deletions to only include the unique gene IDs in each cluster (*i.e.*, if the same gene was affected by multiple different deletions, we did not consider that gene for further analysis). For all of the genes with nonsynonymous mutations with high or moderate impacts, a gene ontology (GO) enrichment analysis was performed using the R package topGO (Alexa and Rahnenfuhrer 2020), and a Fisher’s Exact test using a weighted model was used to calculate significance. As a comparison, we also performed a GO enrichment analysis on *all* affected genes.

### Data availability

The vcf file with all called of SVs, which was filtered out only imprecise calls and pair end less than 3, was uploaded to Dryard Digital Repository (available at https://datadryad.org/stash/share/X7mka20BtXuACd20QbtenGqS-yoGVfKZOZNQQzfe2B0). The vcf file with no filter is available upon request. All the calculations and statistical analysis was conducted in R with custom code (all R scripts are available at https://github.com/skittikun/SVs_in_sorghum_BAP). Supplementary Material is available at figshare: https://doi.org/10.25387/g3.14525475.

## Results

### Genomic distribution of structural variations

The total number of SVs identified in the 347 genotypes prior to filtering was 1,468,572, but after applying all quality, size, and missing data filters, there were a total of **24,648** SVs, including **22,359** deletions, remaining for analysis (Table 1). The majority of the raw SV calls (nearly 1.3 million) were removed after filtering out imprecise calls and sites with an undetermined break point (BND). Of the remaining 116,499 sites, over three quarters of them (78.8%) had missing data for more than 20% of the 347 samples. Out of the final set of 24,648 high-quality and high-confidence SVs, there were 8170 with a MAF >5%, which is equivalent to occurring in at least 18 of the 347 samples. The SV sizes ranged from 51 to 92,311 bp with a median size of 932 bp. Among the SVs with MAF >5%, there were 7766 (95.06%) deletions, 337 (4.12%) duplications, and 67 (0.82%) inversions. Due to limitations of the LUMPY SV caller, insertions could not be detected in this study.

**Table 1:** Numeric summary of SVs along the filter and quality control steps

	No filters	Pair end	Precise	BND	Size less than 100 kb	Missing SV frequency less than 0.2	Minor SV frequency more than 0.05	Min size (bp)	Max size (bp)	Median (bp)
Total	1,468,572	1,451,423	401,999	116,499	103,676	24,648	8,170	51	92,311	932
Deletion	187,099	187,097	105,499	105,499	97,165	22,359	77,66	51	89,716	956
Duplication	40,423	40,420	10,179	10,179	5,856	2,009	337	74	92,311	267
Inversion	3,412	3,410	821	821	655	280	67	55	88,991	280
BND	1,237,638	1,237,638	285,500							

BND, Break-end: generic rearrangement of unknown architecture.

Since deletions were the type of SV that we could call most frequently and with the highest confidence given our dataset, we focused most of our downstream analyses on deletions only.

**Figure 1:**
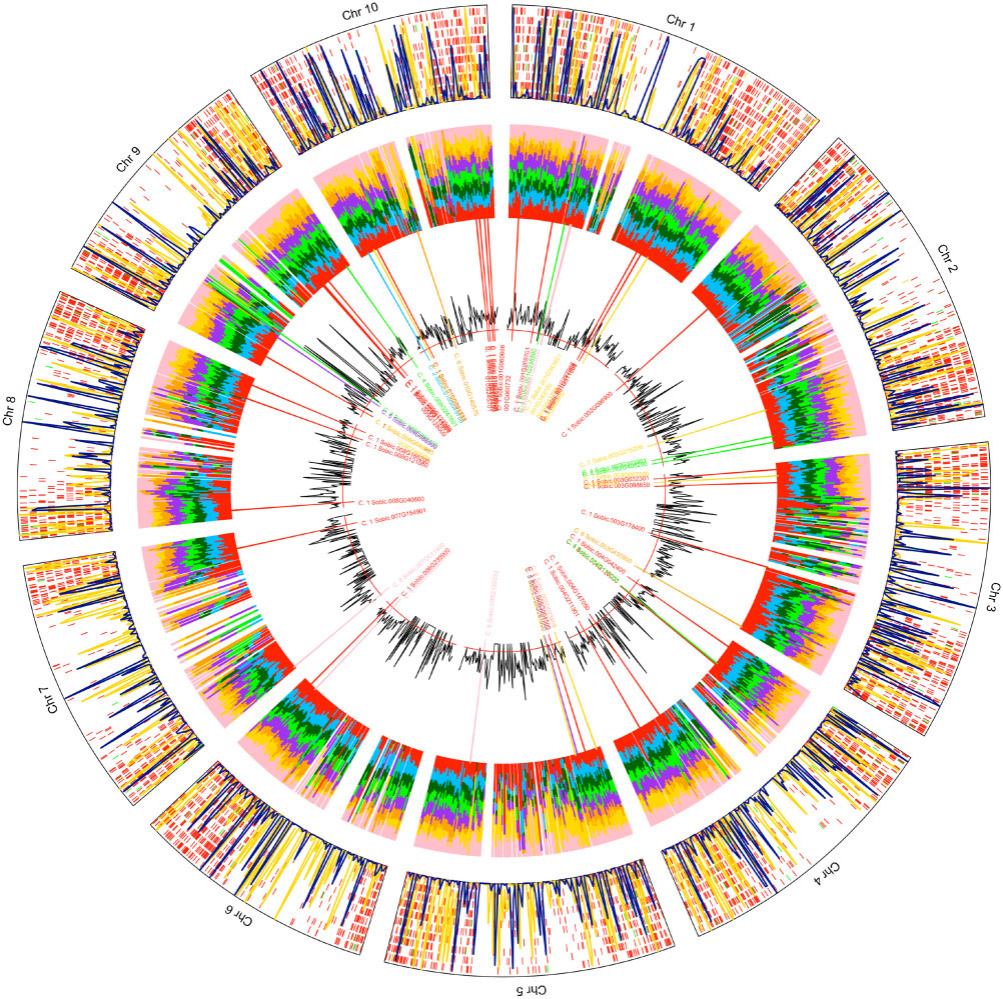
Genome-wide pattern of SVs among 347 sorghum genotypes. The outer ring shows the overall distribution of SVs, where each dash represents a single SV. Red dashes represent deletions, yellow dashes represent inversions, and green dashes represent duplications. The blue and yellow lines in the outermost ring show the π of deletions and SNPs, respectively, in 500 kb sliding windows. The middle ring shows the relative abundance of deletions in 500 kb sliding windows within each of the five groups identified by k-means clustering: group 1 (red), 2 (light blue), 3 (dark green), 4 (light green), 5 (purple), 6 (orange), 7 (yellow), and 8 (pink). The innermost ring is the significance [-log(p)] value of each 500 kb window, indicating whether or not the relative abundance in the window is significantly different from the expected distribution of SVs among the eight groups. The red line shows the Bonferroni corrected significance threshold. The cluster-specific deletions are labeled with the cluster colors on their starting positions.

The distribution of deletions across all 10 sorghum chromosomes ranged from 7.24% on chromosome 7 to 14.61% on chromosome 1 (Table 2). Most of the chromosomes had an average deletion density of 1.11–1.40 deletions/100 kbp. Chromosome 7 had the lowest density, with 0.86 deletions/100 kbp, and chromosome 1 had the highest density, which was nearly double the frequency of deletions on chromosome 7 at 1.40. The majority of deletions detected in this study were located on the ends of the chromosome arms (Figure 1 outermost ring), which are regions that are typically higher recombination and more gene rich. Despite this, three quarters of the deletions (5863) were located outside of any gene regions. Only 1903 (24.50%) deletions were within genes, and these were distributed relatively evenly across the chromosomes, similar to the pattern of intergenic deletions: ranging from 7.67% on chromosome 7 to 15.97% on chromosome 1. Only 641 (8.25%) of the deletions that aligned with gene regions occurred with the CDS.

**Table 2:** Deletion size, number, and overlap to genes or CDS in each chromosome

Chromosome	Number of deletions	Number of deletions per 100 kbp	Min size	Max size	Numbers of deletions within genes	Numbers of deletions within CDS	Numbers of deletions affecting genes from snpEff
Chr01	1135 (14.61)	1.4	51	76,339	304 (15.97)	91 (14.2)	124 (16.17)
Chr02	955 (12.3)	1.23	59	73,918	237 (12.45)	84 (13.1)	68 (8.87)
Chr03	889 (11.45)	1.2	52	84,845	224 (11.77)	74 (11.54)	94 (12.26)
Chr04	744 (9.58)	1.08	53	87,736	178 (9.35)	57 (8.89)	75 (9.78)
Chr05	821 (10.57)	1.14	53	73,942	180 (9.46)	74 (11.54)	103 (13.43)
Chr06	670 (8.63)	1.09	56	89,716	151 (7.93)	45 (7.02)	60 (7.82)
Chr07	562 (7.24)	0.86	54	72,289	146 (7.67)	48 (7.49)	54 (7.04)
Chr08	662 (8.52)	1.06	55	84,593	159 (8.36)	56 (8.74)	50 (6.52)
Chr09	654 (8.42)	1.1	55	67,250	164 (8.62)	56 (8.74)	75 (9.78)
Chr10	674 (8.68)	1.1	55	86,591	160 (8.41)	56 (8.74)	64 (8.34)
Total	7,766 (95.06)				1,903 (24.50)	641 (8.25)	767 (9.88)

The numbers in parenthesis were percentages from total of each chromosome or from 7,766 deletions.

Among the 7766 filtered, high confidence, and MAF >5% deletions we identified, we found a 7411 bp deletion on chromosome 9 between positions 59145680 and 59153091. The location of this mutation matched exactly with a previously identified indel located within the promoter region of the flowering time gene *Elf3* (Li *et al.* 2018). We also observed that accession “PI 655996” did *not* have a deletion, which is consistent with what was previously reported. We were also able to confirm the presence of eight other known sorghum SVs in our *unfiltered* results, though many of these were excluded from further analyses due to an excess of missing data or a low MAF among the accessions we examined (see Supplementary Table S2 for details).

The genome-wide diversity (π) of deletions was slightly higher (1.2X) than the average diversity at SNP sites. In addition, the SNP and deletion diversity measures were *not* correlated at a spatial scale when comparing the two within 500-kb windows (*r* = 0.052 *P*-value = 0.06088) (Figure 1; outermost ring).

### Frequency spectrum and patterns of linkage disequilibrium

**Figure 2:**
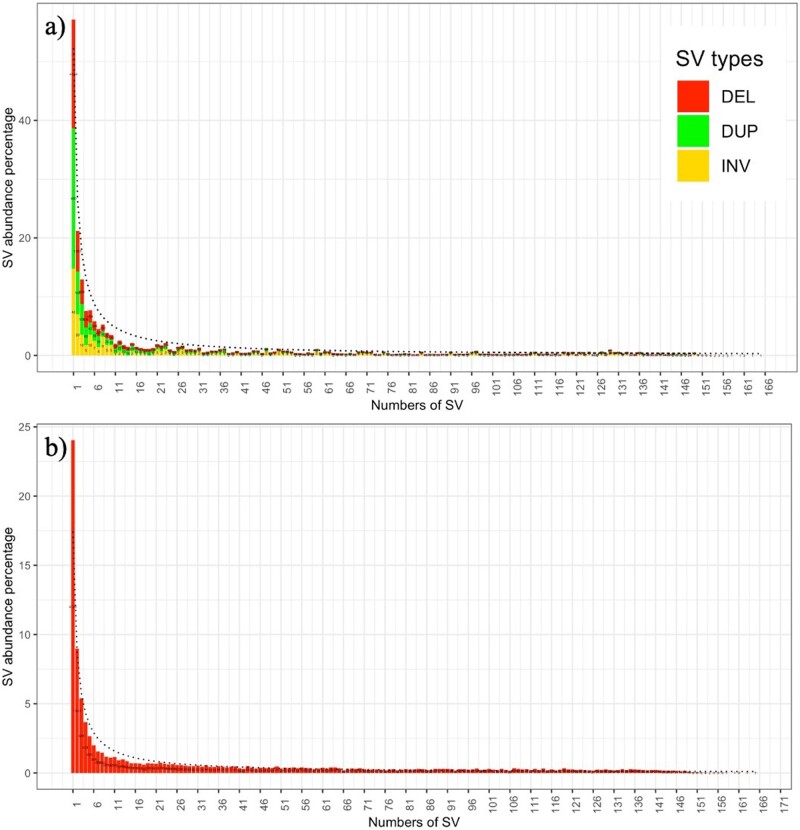
Global site frequency spectrum of SVs. (A) All types of SVs identified in the data. Yellow bars indicate inversions, green bars indicate duplications, and red bars indicate deletions. The dotted black line shows the expected distribution under neutral evolution. (B) Deletions only.

To determine if SVs in sorghum were collectively neutral or potentially under selection, the observed and expected site frequency spectra were calculated and compared for the complete set of filtered 24,648 SVs (Figure 2). The observed frequency spectrum of SVs appeared to follow the same pattern as the expected distribution, regardless of the type of SV (Supplementary Figure S2), strongly suggesting that SVs as a whole are evolving under neutral processes in sorghum.

This pattern also holds true when considering only the deletions (Figure 2B).

**Figure 3:**
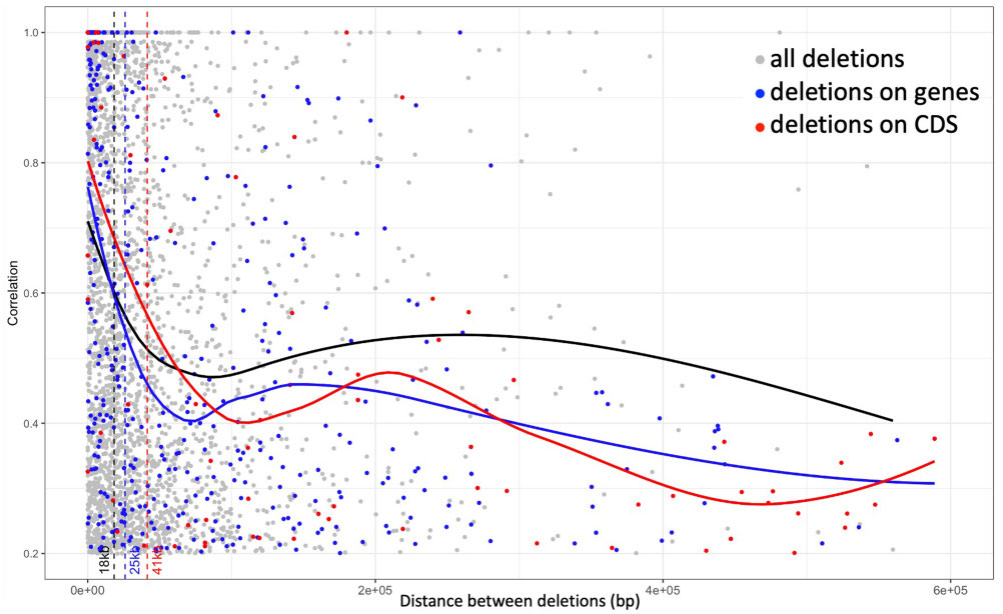
Genome-wide decay of LD between deletions among 347 genotypes. LD was calculated as the correlation coefficient (*R*^2^). Blue, red, and black curves represent a moving average calculated in 100 bp sliding windows. The black curve shows the decay of LD for deletions (7766); the blue curve shows the decay for only deletions aligned within genes (1903); the red curve shows the decay for only deletions aligned within CDS regions (641). Vertical dashed lines indicate the point where the average LD decayed to half of its original value (LD_1/2_).

The genome-wide pattern of LD between all pairs of 7766 deletions with MAF >5% showed that LD decayed to half of its maximum value within 18 kb (Figure 3). However, pairwise LD between deletions located exclusively within gene regions decayed at a slightly slower rate with LD_1/2_ = 25 kb, and the LD between deletions occurring within the CDS decayed at the slowest rate with LD_1/2_ = 41 kb.

### Clustering of deletions with geographic origins and races

**Figure 4:**
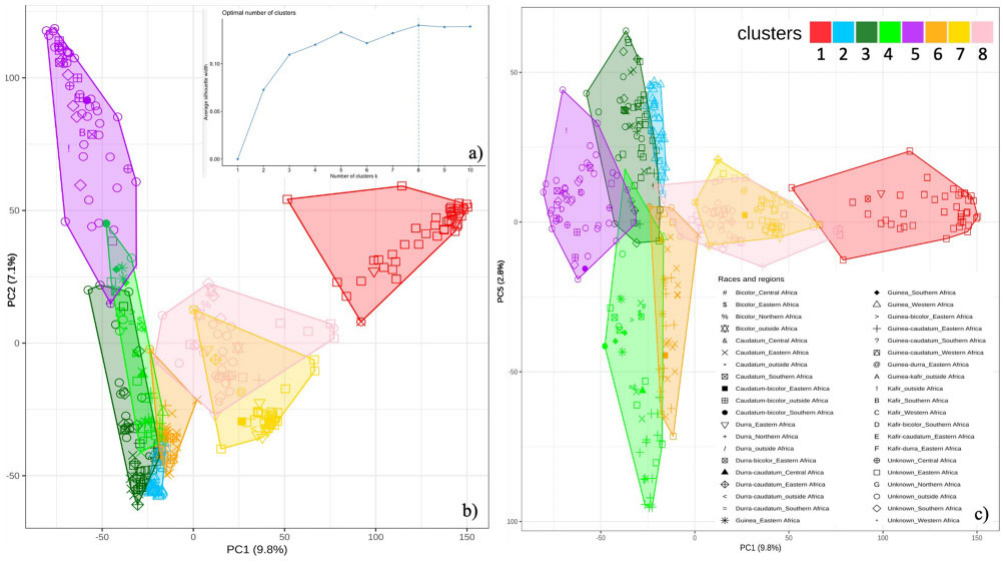
K-means clustering of SNP data into eight groups. (A) Average silhouette width for different values of *K*. The vertical dashed line indicates the *k*-value with the highest silhouette width (*k* = 8). (B, C) PCA plot for the PC1-PC2 and PC1-PC5. Colors indicate the eight groups identified as the optimal number of clusters. Shapes indicate the races and the regions of origin. Percentages indicate the percent variation explained by each PC.

**Figure 5:**
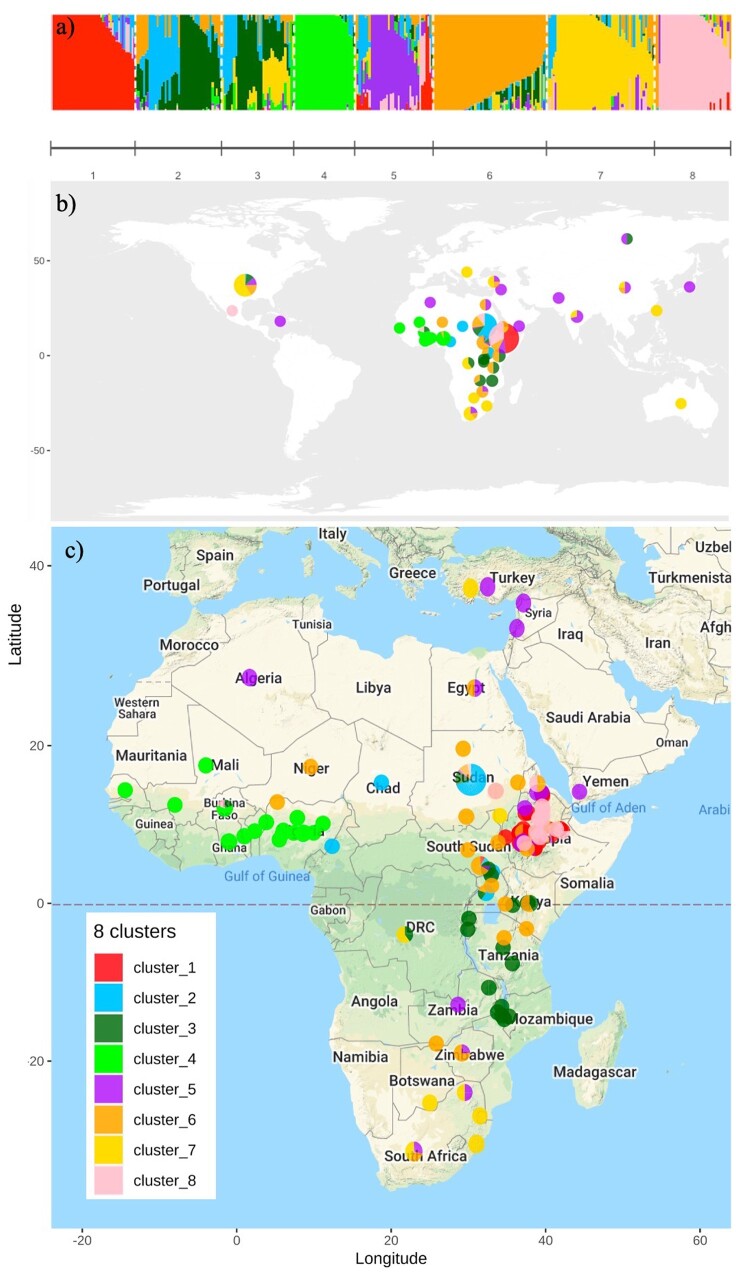
ADMIXTURE results with *K* = 8. (A) Cluster membership for each individual in the eight clusters identified by both ADMIXTURE and k-means clustering. (B) The geographic distributions of populations sampled in this study, with colors corresponding to the cluster membership of each population. (C) The distribution and cluster membership of African populations in this study.

By varying the number of groups, the highest silhouette width in the k-means clustering analysis from SNPs suggested that the genotypes were grouped into eight clusters (Figure 4A). Principal components 1 and 2 (PC1 and PC2) clearly separated clusters 1, 3, 5, 6, and 7 (Figure 4B), but clusters 2 and 4 appeared to overlap with cluster 3, while cluster 7 appeared to overlap with cluster 8. PC5 showed the separation of clusters 2 and 4 from cluster 3 (Figure 4C). PC3 and PC8 separated clusters 7 and 8 (Supplementary Figure S3). The results from ADMIXTURE followed the same pattern, with some individuals showing a mixture of membership in clusters 2 and 3 (Figure 5A).

Cluster 1 (the red cluster in Figures 4 and 5) was almost exclusively comprised of individuals from Ethiopia (42 of 43 genotypes) (Figure 5C and Table 3), all of which were types of unknown or undefined race (Table 4). The majority of individuals assigned to cluster 2 (light blue) were from Sudan (37 of 44 genotypes), and many of these were either caudatums or guinea-caudatum hybrids (15 and 17 genotypes, respectively). Cluster 3 (dark green) contained several different races including guinea, caudatum, and guinea-caudatum hybrids, but all individuals in this cluster originated from regions of Eastern Africa. Cluster 4 (light green) corresponded solely to genotypes from West Africa, the majority of which were guinea types (27 of 31 genotypes). Cluster 5 (purple) corresponded mostly to genotypes with origins outside of Africa (24 of 40) and unknown race (32 of 40 genotypes). Like cluster 3, cluster 6 (orange) also consisted genotypes from East Africa (38 of 58 genotypes), and this cluster also showed some overlap in geography with cluster 3, although it did not extend as far to the south (Figure 5). Cluster 7 (yellow) contained more than half of the individuals originating from the United States (31 out of 53 genotypes), which were mostly unknown or undefined race. This cluster also contained a number of individuals from Southern Africa. Lastly, cluster 8 (pink) contained almost exclusively genotypes from Ethiopia (37 of 39 genotypes). Unlike cluster 1, however, which consisted only of Ethiopian individuals with unknown race, cluster 8 contained many durras and durra hybrids (13 out of 39).

**Table 3:** Origin countries of each genotypes separated into eight clusters from k-mean clustering

Countries of origin	Regions	Cluster 1	Cluster 2	Cluster 3	Cluster 4	Cluster 5	Cluster 6	Cluster 7	Cluster 8	Total
Ethiopia	Eastern Africa	42	—	—	—	8	8	—	29	87
Sudan	Eastern Africa	—	37	7	—	1	10	1	6	62
United States	North America	—	1	6	—	6	9	31	—	53
Nigeria	Western Africa	—	—	—	16	—	1	—	—	17
South Sudan	Eastern Africa	1	1	2	—	1	10		—	15
South Africa	Southern Africa	—	—	—	—	3	2	8	—	13
Kenya	Eastern Africa	—	—	5	—	—	5	—	—	10
India	Asia	—	—	—	—	5	—	2	—	7
Uganda	Eastern Africa	—	3	2	—	—	2	—	—	7
Burkina Faso	Western Africa	—	—	1	3	—	—	—	1	5
Congo	Central Africa	—	—	2	—	—	—	3		5
Ghana	Western Africa	—	—	—	5	—	—	—	—	5
Malawi	Southern Africa	—	—	5	—	—	—	—	—	5
China	Asia	—	—	—	—	2	—	1	—	4
Eritrea	Eastern Africa	—	—	—	—	—	1	1	2	4
Tanzania	Eastern Africa	—	—	2	—	—	2	—	—	4
Turkey	Asia	—	—	—	—	1	1	2	—	4
Zimbabwe	Southern Africa	—	—	—	—	1	3	—	—	4
Pakistan	Asia	—	—	—	—	3	—	—	—	3
Togo	Western Africa	—	—	—	3	—	—	—	—	3
Zambia	Southern Africa	—	—	2	—	—	1	—	—	3
Egypt	Northern Africa	—	—	—	—	1	1	—	—	2
Mali	Western Africa	—	—	—	2	—	—	—	—	2
Russia	Europe	—	—	1	—	1	—	—	—	2
Syria	Asia	—	—	—	—	2	—	—	—	2
Taiwan	Asia	—	—	—	—		—	2	—	2
Yemen	Asia	—	—	—	—	2	—	—	—	2
Algeria	Northern Africa	—	—	—	—	1	—	—	—	1
Australia	Australia	—	—	—	—	—	—	1	—	1
Benin	Western Africa	—	—	—	1	—	—	—	—	1
Botswana	Southern Africa	—	—	—	—	—	—	1	—	1
Burundi	Central Africa	—	—	1	—	—	—	—	—	1
Cameroon	Central Africa	—	1	—	—	—	—	—	—	1
Chad	Central Africa	—	1	—	—	—	—	—	—	1
Eswatini	Southern Africa	—	—	—	—	—	—	1	—	1
Jamaica	North America	—	—	—	—	1	—	—	—	1
Japan	Asia	—	—	—	—	1	—	—	—	1
Mexico	North America	—	—	—	—	—	—	—	1	1
Niger	Western Africa	—	—	—	—	—	1	—	—	1
Rwanda	Eastern Africa	—	—	1	—	—	—	—	—	1
Senegal	Western Africa	—	—	—	1	—	—	—	—	1
Serbia	Europe	—	—	—	—	—	—	1	—	1
Total		43	44	37	31	40	58	55	39	347

**Table 4:** Race of each genotypes separated into eight clusters from k-mean clustering

Races	Cluster 1	Cluster 2	Cluster 3	Cluster 4	Cluster 5	Cluster 6	Cluster 7	Cluster 8	Total
Bicolor	—	—	2	—	4	—	—	—	6
Caudatum	—	15	7	—	—	10	2	—	34
Caudatum-bicolor	—	1	1	—	—	—	4	1	7
Durra	1	—	—	—	2	1	—	8	12
Durra-bicolor	1	—	1	—	—	—	—	1	3
Durra-caudatum	—	1	2		1	2	—	2	8
Guinea	—	2	6	27	—	—	—	1	36
Guinea-bicolor	—	—	1	—	—	—	—	—	1
Guinea-caudatum	—	17	8	3	1	3	—		32
Guinea-durra	—	—	—	—	—	—	—	1	1
Guinea-kafir	—	—	—	—	—	—	1	—	1
Kafir	—	—	—	—	—	1	3	—	4
Kafir-bicolor	—	—	—	—	—	—	1	—	1
Kafir-caudatum	—	1	—	—	—	—	—	—	1
Kafir-durra	—	—	—	—	—	—	—	1	1
Unknown	41	7	9	1	32	41	44	24	199
Total	43	44	37	31	40	58	55	39	347

### Cluster-specific deletions and potential impacts on gene functions

At the genome-wide scale, there were significant differences in deletion abundance among the 8 clusters in almost all 500 kb sliding windows (Figure 1). Of the total 7766 deletions, 767 of them were categorized as cluster-specific (Table 5 and Supplementary Table S3). There were 124 cluster-specific deletions that were categorized as nonsynonymous mutations (either high or moderate impact) by snpEff (Supplementary Table S4). The overall proportion of cluster-specific deletions that were nonsynonymous was very similar to the proportion of noncluster-specific deletions that were nonsynonymous (16% of cluster-specific *vs* 17% of noncluster-specific), suggesting that deletions in general play an important role in both standing and regional levels of genetic variation. For this study, we focused on the impact of deletions that were unique to specific geographic locations.

**Table 5:** Abundance of cluster-specific deletions, number of cluster-specific deletions potentially affecting gene functions (annotated with snpEff as high and moderate impacts), and the uniqueness of those genes among clusters

Clusters	Number of cluster-specific deletions	Number of cluster-specific deletions affecting gene functions	Number of affected genes unique among cluster-specific deletions
1	407	36	40
2	21	1	1
3	8	0	0
4	67	6	6
5	14	2	2
6	44	2	2
7	115	9	13
8	91	8	9
Total	767	64	73

There were 73 unique gene IDs associated with moderate or high impact nonsynonymous cluster-specific deletion (Supplementary Table S5). In five out of the eight clusters, we identified, there were a number of genes with functions related to biotic and abiotic stress that had been impacted by a nonsynonymous deletion.

Cluster 1 had a total of 40 genes that were impacted by a nonsynonymous deletion, which was the highest number of any cluster. Many of these genes have been previously linked to local adaptation to both abiotic and biotic factors in other plant species. We found several genes potentially related to drought tolerance that had deletions unique to cluster 1, which is particularly interesting given that cluster 1 is comprised almost entirely of genotypes from Ethiopia. These drought tolerance genes included Sobic.004G042400, a Myb/SANT-like DNA-binding domain protein that showed differential abundance in response to drought in sugarcane (Salvato *et al.* 2019), Sobic.004G183600, a molybdenum cofactor biosynthesis protein whose overexpression was found to confer increased drought tolerance in maize (Lu *et al.* 2013), and Sobic.008G100400, a member of the pentatricopeptide repeat family with critical roles in stress and development in many plant species (Sharma and Pandey 2015). Two genes with cluster 1 specific deletions had putative roles in both drought and salt tolerance. These were Sobic.004G120000, an expansin gene whose overexpression conferred increased drought and salt tolerance in *A. thaliana* (Lü *et al.* 2013), and Sobic.008G040600, bZIP transcription factor domain containing protein with many potential roles in plant abiotic stress (Banerjee and Roychoudhury 2017). There was also a nonsynonymous deletion in Sobic.007G154901, a gene that encoded a bifunctional monodehydroascorbate reductase and carbonic anhydrasenectarin-3 gene, which is a ROS-related protein important for environmental adaptation in sweet potato (Huang *et al.* 2008). Another interesting deletion was found in Sobic.010G245701, a gene encoding an anthocyanidin 5,3-O-glucosyltransferase. Even though this gene has not been linked to a specific stress response, it has been shown to be important in latitudinal adaptation in *Pinus sylvestris* (Oleszek *et al.* 2002).

Cluster 1 also exhibited a handful of nonsynonymous deletions in genes related to biotic stress tolerance. These included Sobic.006G239900, which encoded a homolog of the Cf-4A gene that is essential for leaf mold resistance in tomato (Kruijt *et al.* 2005), Sobic.010G228800, a leucine rich repeat (LRR) domain containing gene with roles in disease resistance in many plants (Goff and Ramonell 2007), and Sobic.002G096900, which encodes *autophagy-related protein 3* and has been shown to be important for resistance to local viral diseases in cotton (Boya *et al.* 2013; Ismayil *et al.* 2020).

Cluster 4, which corresponded to the West African guinea-type sorghums, had a total of 6 genes affected by cluster-specific nonsynonymous deletions, 3 of which have been previously linked to adaptive phenotypes in other plant species. These genes included an *xa1* gene (Sobic.004G117000) which is known to confer broad-spectrum pathogen resistance in rice (Ji *et al.* 2020), a late embryogenesis abundant (LEA) hydroxyproline-rich glycoprotein related gene (Sobic.002G363901) with abiotic-stress induced expression in sweet oranges (Pedrosa *et al.* 2015), Sobic.001G238500, a gene encoding a UDP-glucoronosyl and UDP-glucosyl transferase, which was related to flavonoid synthesis and connected to latitudinal adaptation in *P. sylvestris* (Oleszek *et al.* 2002).

There were only two genes affected by cluster-specific deletions cluster 5, which was the most geographically widespread cluster. One of the genes with a nonsynonymous deletion was Sobic.005G081000, a member of the pentatricopeptide gene family that has been linked to drought tolerance in a number of plant species (Sharma and Pandey 2015).

Cluster 6 only had one unique nonsynonymous deletion with a potential role in local adaption. This deletion occurred within Sobic.003G435900, a gene that encodes a homolog of the disease resistance protein RPS2 in *A. thaliana* (Bent *et al.* 1994).

Cluster 8, which like cluster 1, was localized almost entirely to Ethiopia, had one unique deletion affecting Sobic.001G252600, which encodes an AAA-type ATPase gene that is responsible for salt-stress tolerance in the halophyte ice plant (*Mesembryanthemum crystallinum*) (Jou *et al.* 2006).

Finally, there were three clusters, clusters 2, 3, and 7, which did not have any nonsynonymous cluster-specific deletions that occurred within genes with clear connections to local adaptation. Although cluster 2 had one unique gene ID impacted by a cluster-specific deletion, this gene (Sobic.010G049700) had no annotated function or homologs in other plant species. In cluster 3, *none* of the cluster-specific deletions that we identified caused a nonsynonymous change in any gene. In cluster 7, there were 11 unique genes affected by cluster-specific deletions, but none of these genes had any known functions in abiotic or biotic stress response. This may be a result of the fact that the genotypes assigned to cluster 7 were also quite geographically widespread (Figure 5), and included individuals from southern Africa, the US, Australia, and Asia.

### Gene ontology analysis of genes affected by deletions

**Figure 6:**
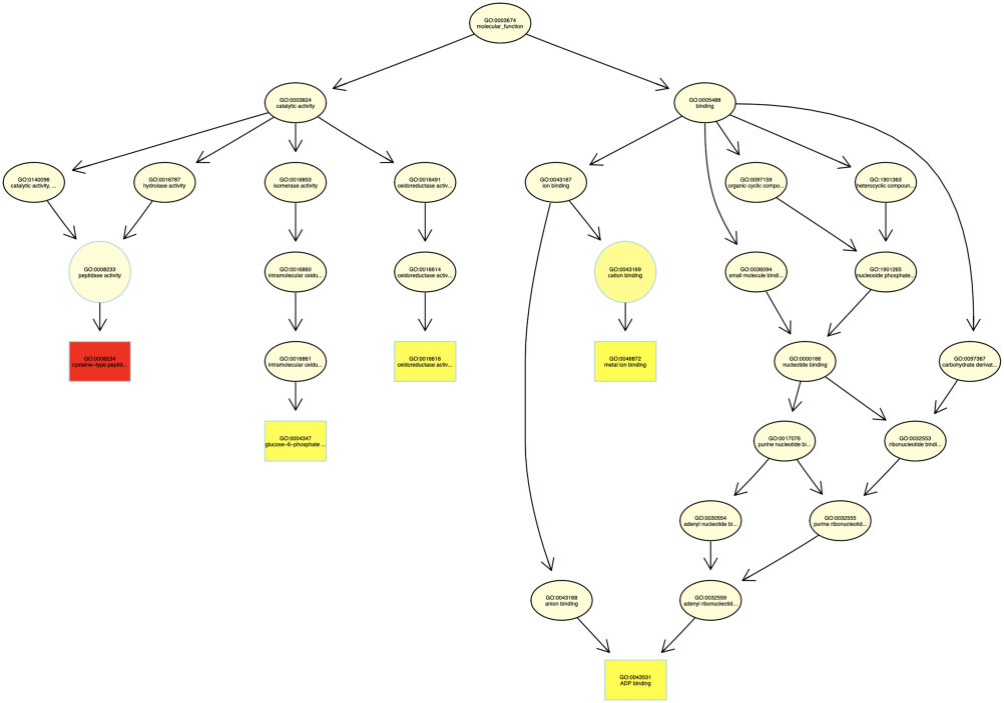
TopGO analysis of all genes affected by deletions based on high or moderate impact from snpEff result. Colors indicate the significance of enrichment for a particular GO term, with red indicating the most significantly enriched category.

A GO analysis of all genes affected by deletions (both cluster-specific and nonspecific) determined from snpEff revealed 8 significant GO terms (Supplementary Table S6). Cysteine-type peptidase activity, metal ion binding, and ADP binding were the three most significant GO terms for genes affected by deletions (Table 6 and Figure 6). Among just the cluster-specific genes with deletions, there were two significantly enriched GO terms: (1) 4 iron, 4 sulfur cluster binding and (2) 3-beta-hydroxy-delta5-steroid dehydrogenase activity (Supplementary Table S7 and Supplementary Figure S4). These two terms were also present in the analysis of noncluster-specific deletions, but neither was statistically significant (*P* = 0.132 for 4 iron, 4 sulfur cluster binding, and *P* = 0.066 for 3-beta-hydroxy-delta5-steroid dehydrogenase activity). ADP binding, which was a significantly enriched GO term among the noncluster-specific deletions, was also found in the cluster-specific analysis but slightly above the threshold of significance (*P* = 0.074).

**Table 6:** Enriched gene ontology of SVs on CDS showed top 10% GO ID from Fisher's exact test

GO.ID	Term	Annotated	Significant	Expected	Rank	*P*-value from Fisher's exact test
GO:0008234	Cysteine-type peptidase activity	121	16	1.7	1	1.2e-11
GO:0046872	Metal ion binding	1,867	43	26.24	3	0.00061
GO:0043531	ADP binding	302	12	4.25	5	0.00117
GO:0016616	Oxidoreductase activity, acting on the CH-OH group of donors, NAD or NADP as acceptor	108	6	1.52	8	0.02753
GO:0004347	Glucose-6-phosphate isomerase activity	2	1	0.03	11	0.02792
GO:0003935	GTP cyclohydrolase II activity	3	1	0.04	12	0.04158
GO:0008686	3,4-dihydroxy-2-butanone-4-phosphate synthase activity	3	1	0.04	13	0.04158
GO:0030247	Polysaccharide binding	96	4	1.35	14	0.04632
GO:0008408	3'-5' exonuclease activity	26	2	0.37	15	0.05123
GO:0051015	Actin filament binding	4	1	0.06	16	0.05506
GO:0003854	3-beta-hydroxy-delta5-steroid dehydrogenase activity	30	2	0.42	18	0.06614
GO:0005094	Rho GDP-dissociation inhibitor activity	5	1	0.07	21	0.06835
GO:0005337	Nucleoside transmembrane transporter activity	5	1	0.07	22	0.06835
GO:0016620	Oxidoreductase activity, acting on the aldehyde or oxo group of donors, NAD or NADP as acceptor	31	2	0.44	25	0.07007
GO:0010333	Terpene synthase activity	43	2	0.6	35	0.12222
GO:0030599	Pectinesterase activity	43	2	0.6	36	0.12222
GO:0051539	4 iron, 4 sulfur cluster binding	10	1	0.14	38	0.13205
GO:0015018	Galactosylgalactosylxylosylprotein 3-beta- glucuronosyltransferase activity	11	1	0.15	40	0.14426

## Discussion

In this study, we identified a total of 24,648 SVs, including 22,359 deletions, among 347 diverse sorghum genotypes. This number was similar, although slightly lower than, previous observations by Zheng *et al.* (2011). The difference in the number of observations is likely due to differences in sequencing depth, detection programs, and filtering based on the sizes of deletions. Because our study was based on Illumina short-read sequencing with an average depth of ∼30X, we are almost certainly underestimating the total number of SVs in sorghum, especially in highly heterochromatic regions, due to technological limitations in SV calling software; nevertheless, the deletions that we report here are all high confidence calls that are informative about the evolutionary history and impact of deletions in sorghum.

The genetic diversity (π) of deletions was slightly higher than genetic diversity of SNPs in sorghum, which is different from a previous study in rice (Kou *et al.* 2020). However, we observed a lack of correlation between SNP diversity and deletion diversity when comparing the two types of mutations in sliding windows across the genome, which was also the case in rice (Kou *et al.* 2020).

The distribution of our deletions was high in high recombination regions and equally distributed across chromosomes as expected (Campbell *et al.* 2014; Miles *et al.* 2016; Rowan *et al.* 2019). LD among deletions decayed to half of its starting value within roughly 18 kb, and the overall extent of LD observed between deletions fell within the ranges that have been previously observed in sorghum (20–150 kb) (Mace *et al.* 2013; Morris *et al.* 2013). Although many of the deletions were located near or even within genes, the overall results from the site frequency spectrum strongly suggested that the genomewide pattern of deletions in sorghum was shaped by neutral evolution. This is different from studies of some other plant species, such as poplar, where a genomewide survey of SVs suggested that most variants were under the influence of positive selection (Sun *et al.* 2015). However, another study in inbred *Mimulus guttatus* (Flagel *et al.* 2014) found that large indels were under neutral or purifying selection, and other studies have also found that SVs, even those potentially affecting gene functions, might be evolving neutrally. For example, in the NBS-LRR disease resistance genes in plants, where we observed a number of SVs, there is a high rate of presence/absence variation and gene copy turnover, even in the absence of pathogens (Baumgarten *et al.* 2003; Meyers *et al.* 2005).

We used k-mean clustering to group genotypes solely based on SNPs. When the clustering results were overlaid with known location and race data for each genotype, the pattern of clustering clearly corresponded to geographic origin and race. The most homogenous group was cluster 4, which almost exclusively contained guinea sorghums from West Africa. This region is more humid than the other geographic areas, and it is well known that guinea genotypes have undergone local adaptation to this wet region (De Wet *et al.* 1976). Clusters 2 and 6 both contained many caudatum and guinea-caudatum hybrids, with the majority (80%) of the genotypes in cluster 2 originating from Sudan, while the genotypes in cluster 6 had a slightly wider geographic dispersal across central/Eastern Africa. Together, the geographic distribution of these two clusters matches the known distribution of caudatum types (Venkateswaran *et al.* 2019). The East African country of Ethiopia by itself contained two different clusters of genotypes: cluster 1, which was predominantly comprised of individuals of unknown race, and cluster 8, which was comprised of durras and durra-hybrid types (Figure 5C, Tables 3 and 4). This area is known to be near the center of origin for domesticated sorghum, and it has previously been observed that the topography and climate variation present in Ethiopia has contributed to the diversity of sorghum in that region (Stemler *et al.* 1977; Doggett 1991). Cluster 5 also contained a number of durra and durra hybrid individuals, but in addition to Ethiopia, this cluster also encompassed the Arabian Peninsula, India, and Asia. Taken together, the clustering patterns observed in clusters 5 and 8 perfectly fit the known history of the durra race (Venkateswaran *et al.* 2019). Clusters 3 and 7 were the most heterogeneous in terms of racial types, but both still showed a clear correspondence with known geographic origins (Figure 5).

Because deletions, especially large deletions like the ones we investigated in this study, have the potential to affect gene function and thereby potentially alter phenotypes, we looked more closely at deletions that were uniquely found in a specific geographic cluster to see whether or not any of them may be playing a role in local adaptation. We found that many of the genes with a nonsynonymous cluster-specific deletion had functions relating to biotic (viral and bacterial pathogen) and abiotic (drought, salt, and latitudinal gradient) responses in other plant species (Supplementary Table S3).

For instance, in cluster 4, which corresponded to the West African group, one of the genes affected by a deletion was *xa1*, which studies in rice have shown to be involved in bacterial resistance as an R gene (Yang *et al.* 2018; Ji *et al.* 2020). Local pathogen pressure can be a strong driver of rapid adaptation (Simms and Triplett 1994; Endara *et al.* 2017), so this deletion could be important for maintaining fitness in this particular group of sorghums.

Cluster 1, which was comprised of genotypes almost exclusively collected from sites within Ethiopia, had by far the largest number of genes affected by cluster-specific deletions. There were three genes that had disease resistance functions. *Cf-4A*, an R gene important for resistance against the fungal disease caused by *Cladosporium fulvum* in tomato, had a 97 bp deletion unique to this cluster causing a frameshift mutation, which could suggest that this particular host-pathogen interaction is not as important in this region as it is in the other geographic locations (Kahlon *et al.* 2020). This cluster also had a 141 bp deletion predictably causing exon loss variant in *LRR*, which is well-known for disease resistance (Goff and Ramonell 2007). Lastly, *autophagy-related protein 3*, a gene conferring viral disease resistance in cotton, had a 360 bp deletion (Boya *et al.* 2013; Ismayil *et al.* 2020). This deletion was also annotated as a frameshift mutation, which could potentially disrupt the function of viral resistance within the cluster 1 genotypes.

Meanwhile, cluster 6 also had one gene with a function involved in disease resistance that was affected by a cluster-specific deletion. RPS2, which has been shown to play a role in local adaptation in *A. thaliana* (Bent *et al.* 1994), had a very large 4.5 kbp deletion that was unique to this cluster.

In addition to biotic stresses, there were also 11 cluster-specific deletions impacting genes with functions related to abiotic stresses. Most of them (8 of 11) appeared to be affecting genes related to drought tolerance. Drought is one of the most challenging stress factors driving local adaptation in plant species, and is also of particular importance to continued crop improvement in the face of climate change (Gimeno *et al.* 2009; Xia *et al.* 2010; Barton *et al.* 2020). Cluster 1 had the most cluster-specific deletions related to drought tolerance. The size of these mutations ranged from an 80 bp deletion in a molybdenum cofactor biosynthesis protein 1 to a 3.6 kbp deletion in a Myb/SANT-like DNA-binding domain-containing protein, two genes that have been found to be differentially expressed in response to drought in maize and sugarcane respectively (Lu *et al.* 2013; Salvato *et al.* 2019).

Almost all of the deletions specific to cluster 1, which affected drought tolerance genes, were annotated as exon loss variants, indicating that these mutations likely cause loss of function of these genes for the majority of the genotypes within this cluster. This result is surprising given that cluster 1 is localized to Ethiopia, a region typically associated with more arid climates, and suggests that these genotypes must have alternative drought tolerance mechanisms. Similarly, in cluster 5, which largely corresponded to durra-type sorghums that are often associated with higher drought tolerance, there was also a nearly 2 kbp (1,472 bp) deletion occurring within a pentatricopeptide gene known for drought tolerance in many plant species (Sharma and Pandey 2015).

On the other end of the spectrum were the genotypes from cluster 4, which comprised almost exclusively West African sorghums from higher humidity climates. This cluster had a 1.5 kbp deletion impacting the transcript ablation of LEA hydroxyproline-rich glycoprotein, which was differentially expressed in sweet orange in response to drought (Pedrosa *et al.* 2015). Notably, this gene also showed differential expression in response to salt tolerance, so it may also be playing a role in local adaptation to soil types in this cluster of sorghums.

The functions of multiple different genes that were impacted by cluster-specific deletions together suggest a potential role for deletions in the local adaptation of these sorghum genotypes. Although our global site frequency spectrum suggested that the majority of deletions in sorghum were evolving neutrally, the extent of LD between deletions occurring within CDS regions (LD_1/2_ = 41 kb) was over twice that observed between nongenic deletions (LD_1/2_ = 18 kb). This slower rate of decay could be indicative of selection acting on these gene-impacting deletions (Bouchet *et al.* 2012).

The most significant GO term for genes affected by nonsynonymous deletions was cysteine-type peptidase activity. This enzyme serves multiple functions in plant growth and development, including senescence, nutrient accumulation in seeds, and stress signaling (Grudkowska and Zagdańska 2004). Metal iron binding was the second most significant GO term, which can have an array of functions in heavy metal stress signaling, lipoxygenase activity, and zinc finger binding (Siedow 1991; Ciftci-Yilmaz and Mittler 2008; Hossain and Komatsu 2013). The third most significant GO term was ADP binding protein. These types of proteins function as general signaling proteins, and in some cases also function in disease resistance (Feng *et al.* 2016).

Interestingly, the only two significant GO terms for genes impacted by cluster-specific deletions (4 iron, 4 sulfer cluster binding and 3-beta-hydroxy-delta5-steroid dehydrogenase activity) were both related to stress responses in other plant species (Busi *et al.* 2006; Kappachery *et al.* 2013).

In conclusion, the structural mutations we identified among diverse sorghum genotypes in this study were spread across all 10 chromosomes and showed a strong correlation with known historical patterns of geographic and racial variation in sorghum. While most medium to large deletions appear to be evolving neutrally via genetic drift, there were several cluster-specific nonsynonymous deletions found within genes with functions related to both biotic and abiotic stress response, which suggests that these cluster-specific deletions may play important roles in local adaptation.
